# Self-reporting and measurement of body mass index in adolescents: refusals and validity, and the possible role of socioeconomic and health-related factors

**DOI:** 10.1186/1471-2458-13-815

**Published:** 2013-09-08

**Authors:** Nearkasen Chau, Kénora Chau, Aurélie Mayet, Michèle Baumann, Stéphane Legleye, Bruno Falissard

**Affiliations:** 1 INSERM, U669, Paris F-75014, France; 2 Univ Paris-Sud, Univ Paris Descartes, UMR-S0669 Paris, France; 3 Faculté de médecine, Université de Lorraine, Vandoeuvre-lès-Nancy, Paris, France; 4 University of Luxembourg, INtegrative research unit on Social and Individual DEvelopment (INSIDE), Walferdange, Luxembourg; 5 Institut national des études démographiques, Paris, France; 6 Assistance Publique-Hôpitaux de Paris, Villejuif, Paris F-94804, France

**Keywords:** Body mass index, Self-reporting, Measurement, Validity, Discrepancy, Socioeconomic factors, Heath, Behaviours

## Abstract

**Background:**

Body mass index assessment using self-reported height and weight (BMIsr) can encounter refusals and under/over-reporting while for assessment with measured data (BMIm) refusals can be more frequent. This could relate to socioeconomic and health-related factors. We explored these issues by investigating numerous potential factors: gender, age, family structure, father’s occupation, income, physical/sports activity, subjective weight perception, school performance, unhealthy behaviours, physical/psychological health, social relationships, living environment, having sustained violence, sexual abuse, and involvement in violence.

**Methods:**

The sample included 1559 adolescents from middle schools in north-eastern France. They completed a questionnaire including socioeconomic and health-related data, self-reported height/weight, measured height/weight, and weight perception (participation rate 94%). Data were analysed using logistic regression models.

**Results:**

BMIsr encountered under-reporting (with change in BMI category, 11.8%), over-reporting (6.0%), and reporting refusals (3.6%). BMIm encountered more numerous refusals (7.9%). Reporting refusal was related to living with a single parent, low school performance, lack of physical/sports activity, sustained violence, poor psychological health, and poor social relationships (gender/age-adjusted odds ratios 1.95 to 2.91). Further to these factors, measurement refusal was related to older age, having divorced/separated parents, a father being a manual worker/inactive, insufficient family income, tobacco/cannabis use, involvement in violence, poor physical health, and poor living environment (1.30 to 3.68). Under-reporting was related to male gender, involvement in violence, poor psychological health, and overweight/obesity (as assessed with BMIm) (1.52 to 11). Over-reporting was related to male gender, younger age, alcohol consumption, and underweight (1.30 to 5.35). Weight perception was linked to reporting refusals and under/over-reporting, but slightly linked to measurement refusal. The contributions of socioeconomic and health-related factors to the associations of weight perception with reporting refusal and under/over-reporting ranged from −82% to 44%. There were substantial discrepancies in the associations between socioeconomic/health-related factors and overweight/obesity assessed with BMIsr and BMIm.

**Conclusions:**

BMIsr and BMIm were affected by numerous biases related to vulnerability which were also obesity risk factors. BMIsr encountered under/over-reporting which were related to some socioeconomic and health-related factors, weight perception, and BMIm. BMIm was more affected by refusals than BMIsr due to socioeconomic and health-related factors. Further research is needed.

## Background

The increasing prevalence of overweight and obesity is a public health concern worldwide, originally in high-income countries, now in low- and middle-income countries, especially in urban settings [1]. They are major risk factors for chronic diseases including diabetes, cardiovascular diseases, musculoskeletal disorders, some cancers, depressive symptoms, suicidal behaviours, disability, and premature death [1,2]. Among American adolescents aged 12 to 18 years, the prevalence of overweight increased from 4.4% in 1959–1962 to 6.8% in 1971–1974, reached 10.6% in 1988–1994, and 14.7% in 1999–2000 [3]. Childhood obesity favours obesity, disability, and premature death in adulthood [1]. However, obesity assessment in most population studies has been based on body mass index (BMI) determined from self-reported height and weight (BMIsr) and fairly rarely from measured data (BMIm) [4-7]. One study showed that BMIsr can bias the BMI-mortality association [8].

BMI is the most common indicator for assessing underweight/overweight/obesity in various settings worldwide (clinical, public health, and community-based programs). It is recommended as the appropriate single indicator in children and adolescents [5,6]. But both BMIsr and BMIm are subject to refusals and BMIsr is subject to error, the degree of which, and factors influencing it, remain unclear. One question of interest concerns BMIsr validity. Does BMIsr over or underestimate BMIm? Another concerning both BMIsr and BMIm is whether missingness randomly occurs or not: is it prone to refusal bias such as socioeconomic, health-related, and behavioural features? Most adolescent studies showed that height was more often over-reported and weight under-reported, leading to underestimations of overweight/obesity prevalence [7,9-16]. Although self-reported weight and height are on average only slightly inaccurate, they are unreliable (in terms of magnitude of obesity) in large population subgroups (age and ethnic groups) [17]. A recent literature review reported that the sensitivity of BMIsr for overweight classification ranges from 55% to 76% (one-fourth to one-half of overweight subjects are missed), and overweight prevalence was 0.4% to 17.7% lower using BMIsr vs. BMIm [7]. Reporting/measurement refusals and the roles of the above-mentioned covariates are often neglected, although missing self-reported data ranges from 0% to 23% [7,17] and reaches 14% to 37% in national surveys [17-21]. The BMI threshold values for obesity and underweight, and a wide range of intermediate BMI values defining overweight may be difficult to estimate for some adolescents, especially those with mental difficulties.

Reporting refusal (in anonymous surveys) may be justified by unknown weight/height, but it may be also motivated by denial of perceived underweight, overweight, or obesity. Consequently, it could be explained by potential risk factors for these weight problems, such as low socioeconomic status, lack of physical/sports activity, and poor physical/mental health [1,2,5,22]. To explore possible biases it is thus important, among a wide range of these factors, to identify those influencing reporting refusal. These factors could also motivate measurement refusals, possibly to a greater extent. Importantly, measurement refusal, as a behavioural feature, could relate to certain behavioural traits such as unhealthy behaviours and involvement in violence. Nowadays, drug use is commonly initiated in adolescence [23-26] and can affect cognitive functions [27]. Like unhealthy behaviours and involvement in violence, measurement refusal could be linked to older age, non-intact families, and lower socioeconomic status. Furthermore, because of these potential factors BMIsr over-reporting could not be excluded. It thus appears important to explore it and its covariates. Using BMIsr or BMIm could generate strong bias in population studies on obesity risk patterns/consequences. These problems seem little documented. However, some studies showed that major determinants of reporting error were age, gender, BMIm, and education [4,17].

Subjective weight perception is an adolescent concern and weight dissatisfaction is related to stress, poor quality of life, and suicidal behaviours [2,28-31]. It can affect reported weight as a result of underweight/overweight denial [10,11,16]. Because of “ideal weight” social norms, certain adolescents suffer from their perceived weight, refuse reporting/measurement, and under/overestimate their BMI. Adolescents feeling too fat are more liable to underestimate their BMI [10]. The roles of socioeconomic, health-related, and behavioural factors can be diverse depending on perceived weight (underweight, overweight or obese) in specific social environments. They may also depend on the consequences of weight problems in terms of physical/mental health, social relationships, and vulnerabilities. In contrast, other underweight/overweight adolescents may not suffer from weight-related problems.

This study explored the following key questions: (a) are reporting refusal, under-reporting, over-reporting, and measurement refusal explained by BMIm, weight perception, or socioeconomic, health-related, and behavioural factors?; and (b) do the BMIsr and BMIm categories relate differently to socioeconomic, health-related, and behavioural factors? Knowledge of these patterns could shed light on the selection bias associated with these covariates, which is important when assessing BMI in population studies. This research is original, as most studies have focused on gender, age, education, and ethnic group only [4]. Given the numerous covariates, the results could contribute to the debate on self-reported versus measured BMI. We focused on individuals in middle schools, mostly under 16 years, because school is compulsory in France until 16 and many problems (such as substance use) become more established in late adolescence (16–20 years) and need to be solved sooner. In contrast to national studies in which we have participated [23-26] this study focused on an exhaustive population from a north-eastern urban area in France, so that the subjects were in the same socioeconomic context, free of regional variations.

## Methods

### Study design

The study population comprised all 1,666 students attending three middle schools, two public and one private, chosen as it may reflect a social gradient (various social categories are represented) in the Nancy urban area (410,000 inhabitants), the capital of Lorraine region (2,342,000 inhabitants) in north-eastern France. They cover a relatively large geographical area (comprising 38.000 inhabitants) and comprise 63 classes. The investigation was approved by the Nancy-Metz regional education authority and the *Commission Nationale de l’Informatique et des Libertés* (national review board). Written informed consent was obtained from the respondents.

The study protocol included an invitation to participate transmitted to parents/guardians (April 2010) and data collection (May-June 2010) using an anonymous self-administered questionnaire over a one-hour teaching period, under research-team supervision with teacher assistance (for surveillance, with no influence on the survey). The completed questionnaires were put in a sealed envelop and then in a closed box by the subjects. Two students refused and 89 (5.3%) were absent when the data collection was carried out (for motives independent of the survey). In total 1575 subjects (95%) completed the questionnaires, among which 10 were of unknown gender/age, and 6 were not completed appropriately, leaving 1559 questionnaires (94%) for analysis. This population was close to that of a French school-based population survey in terms of gender, family and health-related factors (Additional file 1).

The questionnaire included demographic and socioeconomic characteristics (age, gender, family structure, parents’ education, occupation, and income), last-trimester school performance, unhealthy behaviours (current alcohol, tobacco, cannabis, hard drug use, and lack of regular physical/sports activity), the WHOQoL-BREF (measuring physical health, psychological health, social relationships, and living environment) [32], violence (violence sustained by the respondent, sexual abuse, and involvement in violence), self-reported height and weight, and directly measured height and weight (as in other studies [7]).

### Measures

#### WHOQoL-BREF

The validated French version was used [33]. It is the short-form of the World Health Organisation Quality of Life questionnaire. The World Health Organisation defines Quality of Life (QoL) as “the individual’s perception of his/her position in life in the context of the culture and value systems in which he/she lives and in relation to his/her goals, expectations, standards, and concerns” [32]. Past research has shown that the WHOQOL-BREF is a good, reliable and valid cross-cultural measure [32]. It had a good internal consistency in its four domains with Cronbach's alpha coefficients of 0.72, 0.70, 0.62, and 0.78, respectively. We used the 25^th^ percentile as a cut-off value (the quartiles are often used for deprivation measures [34]) which appears appropriate for most subjects with health-related issues.

#### Father's occupational category and income

Five categories were considered following the international classification of occupations (ISCO): managers, professionals, and intermediate professionals; craftsmen, tradesmen, and heads of firms; service workers and clerks; manual workers and other occupations; and not working people (unemployed and retired). For perceived income, subjects were asked whether the financial situation of their family was: coping but with difficulties/getting into debt vs. comfortable/well off/earning just enough [35,36].

#### Current alcohol, tobacco cannabis, and hard drug use

Use of these substances was assessed with the questions ‘During the last 30 days’: ‘how many times have you had alcoholic drinks (beer, cider, champagne, wine, aperitif, etc.?’ (None/1-5/6-9/10-29/30+), ‘how many cigarettes a day did you smoke?’ (None/1-4/5-9/10-19/20+ cigarettes/day), ‘on how many occasions have you used any form of cannabis?’ (None/1-5/6-9/10-29/30+), and ‘on how many occasions have you used any form of other illicit drugs (mushrooms, ecstasy, LSD, etc.)?’ (none/1-5/6-9/10-29/30+) [23-26]. These factors were dichotomized (at least once vs. none).

#### Violence sustained by the respondent

This was measured using a 20-item scale (five questions for four localities: in school, school neighbourhood, at home, and elsewhere) [25,26]: ‘During the last 12 months, have you been victim of …?’: hitting, stealing, racket, insult, and racial abuse (yes vs. no). The Cronbach's alpha was satisfactory (0.71), allowing a single score to be calculated. Violence sustained was defined by the presence of at least one item.

#### Involvement in violence

This was measured with a 11-item scale [25,26]: ‘During the last 12 months, have you?’, ‘gotten mixed into a fight at school’, ‘taken part in a fight where a group of your friends were against another group’, ‘belonged to a group starting a fight against another group’, ‘committed insults’, ‘committed racial abuse’, ‘started a fight with another individual’, ‘taken something not belonging to you (in school, in the neighbourhood of school, at home, …’, ‘taken something from a shop without paying for it’, ‘set fire to somebody else's property on purpose’, ‘used any kind of weapon to get something from a person’, or ‘damaged public or private property on purpose’ (yes vs. no). The Cronbach's alpha was satisfactory (0.82), allowing a single score to be calculated. Involvement in violence was defined by the presence of at least one item.

#### Sexual abuse

This was probed for with the question: ‘In the course of your life, have you been a victim of sexual abuse?’ (yes vs. no) [25,26].

#### Weight and height self-reporting and measurement

Self-reports were obtained from two questions: ‘About how much do you weigh without clothes and shoes?’, ‘About how tall are you without shoes?’ [17]. During questionnaire completion and after reporting weight and height, all adolescents were invited to measure their weight and height with the same research-team trained physician. Weight and height measurements were performed in a dedicated area and a second research-team member ensured that peers could not come near. The teachers were not allowed to come close either. Thus, no-one else could read the measurements. Body height was measured with a measuring tape (mounted on a portable stadiometer fixed on the wall). Weight was measured with Scaleman electronic scales (accuracy to 50 grams). Measurements were taken without shoes in a light gown. BMI was defined as weight/height^2^ (kg/m^2^). BMIsr and BMIm values were then categorized into underweight, normal weight, overweight or obese according to the widely used threshold values recommended for male and female French adolescents at different ages [37]. Under and over-reporting were respectively defined as BMIm > BMIsr and BMIm < BMIsr with category changes. Weight perception was assessed by asking if the respondent considered him/herself to be much too thin, a bit too thin, about right, a bit too fat or much too fat [38].

### Statistical analysis

The relationship between self-reported and measured values for height, weight, and BMI as continuous variables was assessed with the Pearson correlation coefficient, intra-class correlation coefficient, and regression models. Their differences were also examined. The associations between reporting refusal, measurement refusal, under-reporting, and over-reporting on the one hand and socioeconomic, health-related, and behavioural factors on the other were evaluated using gender and age-adjusted odds ratios (ORga), odds ratios adjusted for all covariates with 95% confidence intervals (CI). The ORga were also used to examine the associations between feeling too fat or feeling too thin and the same covariates. To study the association between feeling too fat and each outcome variable (reporting refusal, measurement refusal, under-reporting, or over-reporting) three logistic regression models were performed: a basic model measuring their crude association (model 0), BMIm added to model 0 (model 1), and socioeconomic, health-related and behavioural factors added to model 1 (model 2). The contribution of these factors to explaining the association was estimated by the change in the odds ratios (OR) after their inclusion in the model, i.e. the explained fraction calculated by the formula: (OR_model1_–OR_model2_)/(OR_model1_–1) [39]. Positive% values indicate reductions in ORs, and negative% values increases in ORs. The contribution was calculated only if the OR was significant in model 1. The same models were used for feeling too thin. Finally, the ORga and the odds ratios adjusted for all covariates were used to compare to associations of each covariate with BMIsr and BMIm categories. All the analyses were performed using the Stata program (Texas: Stata Corporation 2007).

## Results

The subjects’ characteristics are shown in Table 1. Measurement refusal was twice as common as reporting refusal (7.9% vs. 3.6%). Overall, the distribution of BMIsr and BMIm were close but the frequencies of under and over-reporting were 11.8% and 6.0%. The distribution of subjective weight perception was fairly close to that of BMIsr and BMIm, but feeling much too fat was half as frequent as obesity assessed by BMIsr and BMIm.

**Table 1:** Characteristics of subjects (N = 1,559)

	**Number of subjects**	**% or mean(SD)**
Boys	778	49.9 (1.3)
Age (yr)		
Mean (SD)		13.0 (1.3)
Range (yr)		9.9 to 18.7
Family structure		
Intact	982	63.0 (1.2)
Parents divorced/separated and reconstructed family	391	25.1 (1.1)
Single parent and other situations	186	11.9 (0.8)
Father’s occupation		
Manager, professional, and intermediate professional	595	38.2 (1.2)
Craftsman, tradesman, and head of firm	314	20.1 (1.0)
Service worker and clerk	144	9.2 (0.7)
Manual worker and other occupations	389	25.0 (1.1)
Not working	117	7.5 (0.7)
Insufficient family income	276	17.7 (1.0)
Low school performance (<10/20)	128	8.2 (0.7)
Last-30 day substance use		
Tobacco	174	11.2 (0.8)
Alcohol	549	35.2 (1.2)
Cannabis	87	5.6 (0.6)
Hard drugs	43	2.8 (0.4)
Lack of regular physical/sports activity	182	11.7 (0.8)
Having sustained violence	832	53.4 (1.3)
Victim of sexual abuse	57	3.7 (0.5)
Involvement in violence	927	59.5 (1.2)
WHOQOL ≤25^th^ percentile value		
Physical health	361	23.2 (1.1)
Psychological health	421	27.0 (1.1)
Social relationships	415	26.6 (1.1)
Living environment	392	25.1 (1.1)
Body weight image		
Much too thin	22	1.4 (0.3)
A bit too thin	168	10.8 (0.8)
Right weight	831	53.3 (1.3)
A bit too fat	439	28.2 (1.1)
Much too fat	74	4.8 (0.5)
Non-response	25	1.6 (0.3)
Self-reported body mass index (BMIsr)		
Underweight	39	2.5 (0.4)
Normal weight	908	58.2 (1.2)
Overweight	398	25.5 (1.1)
Obese	158	10.1 (0.8)
Reporting refusal	56	3.6 (0.5)
Measured body mass index (BMIm)		
Underweight	19	1.2 (0.3)
Normal weight	854	54.8 (1.3)
Overweight	397	25.5 (1.1)
Obese	166	10.6 (0.8)
Measurement refusal	123	7.9 (0.7)
Misclassification (with change in category of BMIsr vs. BMIm)		
Under-reporting	157	11.8 (0.8)
Over-reporting	75	6.0 (0.6)

### Differences and relationships between reported and measured height, weight, and BMI

Table 2 shows that among the 1401 subjects (89.9%) with available BMIm and BMIsr, the Pearson correlation coefficient and the intra-class correlation coefficient were high but the kappa coefficients were much lower, showing poor agreement between categorised BMIm and BMIsr. The difference (measured value ‒ reported value) was significantly positive for height, weight, and BMI, with much higher percentages of positive than negative values, except for height among girls. This lack of agreement is detailed in Table 3. There was also poor agreement between weight perception and both BMIm and BMIsr (Additional file 2): 36.8% of underweight, 48.2% of overweight, and 15.2% of obese subjects according to BMIm thought they were the right weight. Among the subjects classified by BMIm as underweight 36.8% felt they were the right weight and 57.9% felt they were a bit too thin. Among those classified by BMIm as obese 15.2% though they were the right weight, 64.2% felt a bit too fat, and only 20.0% felt much too fat.

**Table 2:** Mean difference, Pearson correlation coefficient, intra-class correlation coefficient, and kappa coefficient for reported and measured height, weight, and body mass index (BMI)

	**Measured minus self-reported values**				**Pearson correlation coefficient**	**Intra-class correlation coefficient and 95% CI**	**Non-weighted kappa coefficient (SE) for categorised BMI**	**Regression equation of reported in terms of measured data:regression coefficient and 95% CI**	
	**Mean (SD)**	**<0 (%)**	**Zero (%)**	**>0(%)**				**Slope**	**Constant term**
Boys (N = 708)									
Height (m)	**0.0069** (0.33)	28.8	24.6	46.6	0.96	0.92 (0.89-0.95)	-	1.00 (0.98-1.02)	−0.01 (−0.045-0.025)
Weight (kg)	**1.03** (2.63)	21.5	11.9	66.7	0.98	0.96 (0.95-0.97)	-	**0.96** (0.94-0.97)	**1.06** (0.25-1.88)
BMI (kg/m^2^)	**0.22** (1.21)	32.8	6.6	60.6	0.92	0.90 (0.84-0.96)	0.66 (0.027)	**0.90** (0.87-0.93)	**1.72** (1.16-2.28)
Girls (N = 693)									
Height (m)	0.0016 (0.023)	33.3	28.4	38.2	0.96	0.93 (0.89-0.96)	-	**1.03** (1.01-1.05)	**−0.045** (−0.079- -0.011)
Weight (kg)	**0.81** (2.22)	23.5	11.4	65.1	0.98	0.96 (0.95-0.97)	-	**0.97** (0.96-0.99)	0.61 (−0.19-1.41)
BMI (kg/m^2^)	**0.29** (1.12)	31.8	5.5	62.8	0.94	0.88 (0.83-0.93)	0.75 (0.029)	**0.93** (0.90-0.95)	**1.18** (0.66-1.69)
Total sample (N = 1401)									
Height (m)	**0.0043** (0.29)	31.0	26.5	42.5	0.96	0.96 (0.95-0.96)	-	1.01(0.99-1.02)	−0.014 (−0.038-0.011)
Weight (kg)	**0.92** (2.44)	22.5	11.6	65.9	0.98	0.92 (0.89-0.95)	-	**0.96** (0.95-0.97)	**0.92** (0.35-1.49)
BMI (kg/m^2^)	**0.25** (1.17)	32.3	6.1	61.7	0.93	0.88 (0.85-0.91)	0.70 (0.020)	**0.91** (0.89-0.93)	**1.44** (1.06-1.83)

N: number of subjects.

Bold type: mean value significantly different from zero, slope significantly different from 1, and constant term significantly different from zero (p < 0.05).

**Table 3:** Discrepancy between self-reported and measured body mass indexes (N = 1,559): N (cell%)

		**Measured body mass index (BMIm)**				
		**Underweight**	**Normal**	**Overweight**	**Obese**	**Measurement refusal**
Self-reported body mass index (BMIsr)						
Underweight		14 (0.9)	23 (1.5)	0	0	2 (0.1)
Normal		5 (0.3)	754 (48.4)	92 (5.9)	3 (0.2)	54 (3.5)
Overweight		0	45 (2.9)	280 (18.0)	39 (2.5)	34 (2.2)
Obese		0	9 (0.6)	16 (1.0)	121 (7.8)	12 (0.8)
Reporting refusal		0	23 (1.5)	9 (0.6)	3 (0.2)	21 (1.3)
	N	Underweight	Normal	Overweight	Obese	Refusal
Classification of BMIm among:						
Subjects with known BMIsr	1,503	19 (1.3)	831 (55.3)	388 (25.8)	163 (10.8)	102 (6.8) ^a^
Subjects with reporting refusal	56	0	23 (41.1)	9 (16.1)	3 (5.4)	21 (37.5) ^a^
Classification of BMIsr among:						
Subjects with known BMIm	1,436	37 (2.6)	854 (59.5)	364 (25.3)	146 (10.2)	35 (2.4) ^b^
Subjects with measurement refusal	123	2 (1.6)	54 (43.9)	34 (27.6)	12 (9.8)	21 (17.1) ^b^

N: number of subjects.

Refusal for ^a^ reporting or for ^b^ measurement.

### Relationships of reporting refusal, measurement refusal, BMIsr under-reporting or over-reporting with various factors

Table 4 shows that, based on ORga, reporting refusal was strongly related to having a single-parent, low school performance, lack of regular physical/sports activity, having sustained violence, poor psychological health, poor social relationships, measurement refusal, and weight perception (ORga between 1.95 and 8.63). The factors associated with measurement refusal were: older age, non-intact families, having a father who was a manual-worker or not working, insufficient family income, low school performance, tobacco and cannabis consumption, lack of regular physical/sports activity, involvement in violence, poor physical health, poor psychological health, poor living environment, and feeling much too fat (ORga between 1.31 and 3.60). BMIsr under-reporting was related to male gender, involvement in violence, poor psychological health, overweight and obesity (measured with BMIm), and feeling a bit too fat (ORga between 1.47 and 11.0). BMIsr over-reporting was negatively related to older age (ORga 0.77 per year) and positively related to male gender, alcohol consumption, underweight (measured with BMIm), and feeling too fat (reflecting a tendency towards anorexia) (ORga between 1.78 and 5.35).

**Table 4:** Relationships of reporting refusal, measurement refusal, BMIsr under-reporting or over-reporting with various factors: odds ratio and 95% confidence interval (CI)

	**Reporting refusal**		**Measurement refusal**		**BMIsr under-reporting (resulting in a change in BMI category)**		**BMIsr over-reporting (resulting in a change in BMI category)**	
	**ORga 95% CI**	**ORfm 95% CI**	**ORga 95% CI**	**ORfm 95% CI**	**ORga 95% CI**	**ORfm 95% CI**	**ORga 95% CI**	**ORfm 95% CI**
*N*	*1,559*		*1,559*		*1,326*		*1,244*	
Boys	0.75 0.44-1.28	‒	0.79 0.54-1.14	‒	1.47* 1.05-2.06	‒	2.24† 1.36-3.70	4.22‡ 2.41-7.42
Age (yr)	1.03 0.84-1.28	‒	1.31‡ 1.13-1.52	1.29‡ 1.10-1.50	1.00 0.87-1.14	‒	0.77† 0.64-0.94	0.74† 0.60-0.91
Family structure								
Intact	1.00	1.00	1.00	1.00	1.00	1.00	1.00	1.00
Parents divorced/separated and reconstructed family	1.22 0.64-2.34	‒	1.59* 1.02-2.49	‒	0.94 0.63-1.40	‒	0.83 0.45-1.51	‒
Single parent and other situations	2.45† 1.25-4.82	‒	3.68‡ 2.31-5.85	2.42‡ 1.52-3.85	1.24 0.73-2.10	‒	1.89 0.98-3.63	2.47† 1.24-4.90
Father’s occupation								
Manager, professional, and intermediate professional	1.00	1.00	1.00	1.00	1.00	1.00	1.00	1.00
Craftsman, tradesman, and firm head	1.56 0.74-3.29	‒	1.12 0.63-1.97	‒	0.62 0.38-1.00	0.55* 0.33-0.91	1.66 0.91-3.03	‒
Service worker and clerk	1.33 0.48-3.69	‒	0.70 0.29-1.70	‒	0.81 0.45-1.48	‒	1.55 0.72-3.34	‒
Manual worker and other occupations	1.43 0.70-2.93	‒	1.68* 1.04-2.71	‒	0.78 0.51-1.19	0.53† 0.34-0.83	0.77 0.38-1.57	‒
Not working	2.25 0.90-5.63	‒	3.60‡ 2.03-6.39	‒	0.54 0.24-1.21	0.24† 0.10-0.60	1.60 0.63-4.09	‒
Insufficient family income	1.26 0.66-2.42	‒	1.63* 1.06-2.50	‒	1.15 0.75-1.78	‒	1.25 0.68-2.29	‒
Low school performance (<10/20)	2.91† 1.45-5.86	‒	2.87‡ 1.75-4.70	2.11† 1.24-3.59	1.02 0.52-1.97	‒	1.42 0.58-3.44	‒
Last-30 day substance use								
Tobacco	1.10 0.48-2.51	‒	2.07† 1.29-3.33	‒	0.97 0.55-1.69	‒	0.70 0.27-1.78	‒
Alcohol	0.93 0.52-1.66	‒	1.32 0.90-1.94	‒	1.08 0.75-1.55	‒	1.78* 1.08-2.95	‒
Cannabis	1.32 0.46-3.82	‒	2.19† 1.19-4.02	‒	0.81 0.36-1.83	‒	1.13 0.39-3.28	‒
Hard drugs	0.63 0.09-4.71	‒	2.05 0.88-4.79	‒	0.94 0.32-2.72	‒	0.50 0.07-3.79	‒
Lack of regular physical/sports activity	2.14* 1.11-4.14	‒	2.61‡ 1.66-4.11	1.97† 1.21-3.22	0.97 0.56-1.68	‒	1.12 0.54-2.32	‒
Having sustained violence	1.95* 1.10-3.46	2.68† 1.36-5.29	1.15 0.79-1.68	‒	1.15 0.82-1.62	‒	1.00 0.62-1.60	‒
Victim of sexual abuse	1.45 0.43-4.82	‒	1.64 0.75-3.61	‒	1.71 0.78-3.76	‒	1.48 0.44-5.01	‒
Involvement in violence	1.04 0.59-1.83	0.52* 0.27-0.98	1.79† 1.17-2.73	‒	1.52* 1.05-2.20	1.51* 1.02-2.23	1.24 0.74-2.06	‒
WHOQOL ≤25^th^ percentile value								
Physical health	1.68 0.95-3.00	‒	1.67† 1.12-2.50	‒	1.14 0.76-1.71	‒	1.16 0.64-2.11	‒
Psychological health	2.18† 1.26-3.78	‒	2.07‡ 1.41-3.04	1.60* 1.06-2.40	1.62† 1.12-2.34	‒	1.65 0.97-2.81	‒
Social relationships	1.97* 1.14-3.40	‒	1.32 0.89-1.96	‒	1.08 0.74-1.57	‒	0.68 0.37-1.24	‒
Living environment	1.41 0.79-2.50	‒	2.17‡ 1.48-3.18	‒	1.37 0.94-1.99	‒	0.97 0.54-1.71	‒
Measured body mass index (BMIm)								
Underweight	(1)	‒	(2)	(2)	(1)	‒	5.35† 1.80-15.9	9.96‡ 3.15-31.5
Normal weight (reference)	1.00	1.00	1.00	1.00	1.00	1.00	1.00	1.00
Overweight	0.85 0.39-1.86	‒	(2)	(2)	10.7‡ 6.65-17.3	23.6‡ 12.1-45.8	0.82 0.46-1.46	0.38† 0.20-0.73
Obese	0.68 0.20-2.30	‒	(2)	(2)	11.0‡ 6.39-19.0	25.4‡ 12.3-52.4	(1)	‒
Measurement refusal	7.62‡ 4.03-14.4	8.82‡ 4.74-16.4	(2)	(2)	(2)	‒	(2)	‒
Weight perception								
Much too thin	8.63‡ 2.30-32.4	4.50* 1.13-5.29	1.28 0.28-5.77	‒	2.97 0.93-9.42	9.88‡ 2.49-39.1	1.99 0.25-16.2	‒
A bit too thin	2.97† 1.29-6.86	‒	1.12 0.58-2.14	‒	1.00 0.55-1.84	7.13‡ 3.12-16.3	1.60 0.73-3.51	‒
Right (reference)	1.00	1.00	1.00	1.00	1.00	1.00	1.00	1.00
A bit too fat	1.94 0.96-3.90	‒	1.21 0.78-1.89	‒	2.00‡ 1.37-2.92	‒	3.17‡ 1.84-5.45	7.15‡ 3.97-12.9
Much too fat	4.03† 1.51-10.8	‒	2.41* 1.28-4.90	‒	1.53 0.66-3.55	‒	3.37* 1.21-9.40	19.6‡ 5.98-64.5

*p < 0.05, †p < 0.01, ‡p < 0.001.

BMI: Body mass index; BMIsr: self-reported BMI.

ORga: gender-age-adjusted odds ratio; ORfm: odds ratios adjusted for all factors (full model).

(1) Non computable. (2) Non concerned.

The relationships of under-reporting with overweight and obesity (measured using BMIm) changed little after adjustment for family, father’s occupation and income (ORs 12.29, p < 0.001, 95% CI 7.53-20.07 and 13.21, p < 0.001, 95% CI 7.51-23.22, respectively) nor after further adjustment for health-related and behavioural factors (12.34 and 13.00, respectively). Similarly, the relationship between over-reporting and underweight (measured with BMIm) changed little after further adjustment for family, father’s occupation, and income (adjusted OR 5.00, p = 0.005, 95% CI 1.62-15.44) nor after further adjustment for health-related and behavioural factors (5.21).

Table 4 further shows that logistic models including all factors reveal that the main covariates for reporting refusal were having sustained violence (adjusted OR 2.68), measurement refusal (8.82), and feeling much too thin (4.50); those for measurement refusal were older age (1.29 per year), living with a single-parent (2.42), low school performance (2.11), lack of regular physical/sports activity (1.97), and poor psychological health (1.60); those for BMIsr under-reporting were lower father’s occupational category (between 0.24 and 0.55), involvement in violence (1.51), overweight and obesity (measured with BMIm, 23.6 and 25.4, respectively), and feeling a bit, or much too thin (7.13 and 9.88, respectively); and those for BMIsr over-reporting were male gender (4.22), older age (0.74 per year), living with a single-parent (2.47), being underweight and being overweight (measured with BMIm, 9.96 and 0.38, respectively), and feeling a bit, or much too fat (7.15 and 19.6, respectively).

### Relationships between subjective weight perception and reporting refusal, measurement refusal, BMI under-reporting and BMI over-reporting (vs. correct reporting) and roles of covariates

The relationships between weight perception and various covariates are detailed in Additional file 3. We found that both feeling too fat and feeling too thin were related to a number of factors, and mainly to BMIm, gender, low school performance, and poor psychological health.

As Table 5 shows, feeling too fat was associated with a 2.39 times greater likelihood of reporting refusal and this did not change after controlling for BMIm, but decreased to 1.77 (non-significant, contribution 44%) after controlling for socioeconomic, health-related, and behavioural covariates. Feeling too thin was associated with a 3.43 times greater likelihood of reporting refusal, and a 3.61 times greater likelihood after controlling for BMIm, with a covariate contribution of 10%. Measurement refusal was not associated with either feeling too fat or feeling too thin, both before and after controlling for BMIm and the covariates. Feeling too fat was associated with a 1.80 times greater likelihood of under-reporting and this decreased to 0.56 after controlling for BMIm and to 0.55 after controlling for covariates. Feeling too fat was associated with a 2.61 times greater likelihood of over-reporting and this increased to 5.07 after controlling for BMIm and to 8.40 after controlling for covariates (contribution −82%). Feeling too thin was not associated with over-reporting. It was associated with a 6.32 times greater likelihood of under-reporting after controlling for BMIm, increasing to 7.23 after controlling for covariates (contribution −17%).

**Table 5:** Relationships of weight self-perception with reporting refusal, measurement refusal, BMIsr under-reporting or BMIsr over-reporting (vs. correct reporting) and roles of covariates (N = 1559): odds ratio (OR) and 95% confidence interval (CI)

	**Reporting refusal**		**Measurement refusal**		**BMIsr under-reporting (with change in BMI category; vs. correct reporting)**		**BMIsr over-reporting (with change in BMI category; vs. correct reporting)**	
	**OR**	**%**	**OR**	**%**	**OR**	**%**	**OR**	**%**
	**95% CI**		**95% CI**		**95% CI**		**95% CI**	
**Weight perception**								
Feeling too fat (vs. feeling the right weight)								
Crude OR	2.39†		1.46		1.80‡		2.61‡	
	1.25-4.56		0.97-2.18		1.25-2.58		1.56-4.36	
Model 1	2.38*	100	1.46	‒	0.56†	100	5.07‡	100
	1.18-4.79		0.97-2.18		0.37-0.86		2.91-8.83	
Model 2	1.77	44	1.01	‒	0.55†	2	8.40‡	-82
	0.82-3.81		0.64-1.58		0.35-0.89		4.41-16.0	
Feeling too thin (vs. feeling the right weight)								
Crude OR	3.43‡		1.12		1.23		1.76	
	1.60-7.39		0.61-2.06		0.71-2.12		0.84-3.72	
Model 1	3.61†	100	1.12	‒	6.32‡	100	1.17	‒
	1.63-8.01		0.61-2.06		2.91-13.7		0.52-2.64	
Model 2	3.35†	10	1.05	‒	7.23‡	-17	1.00	‒
	1.45-7.74		0.55-2.00		3.26-16.0		0.41-2.46	

*p < 0.05, †p < 0.01, ‡p < 0.001.

BMI: Body mass index; BMIsr: self-reported BMI.

Model 1: adjusted for measured BMI (except for measurement refusal).

Model 2: with further adjustment for socioeconomic factors and health-related and behavioural factors (Table 4).

% = Reduction (positive%) or increase (negative%) in OR computed with the following formula: (OR _model 1_–HR _model2_)/(OR _model 1_–1).

### Relationships between underweight, overweight, and obesity (vs. normal weight) assessed using self-reported BMI and measured BMIs and various factors

In Table 6, the ORga evidence some discrepancies between BMIsr and BMIm when their links with socioeconomic, health-related, and behavioural factors are examined. Indeed, overweight measured with BMIm and that measured with BMIrs were similarly related to living with a single-parent, having a father being a manual-worker or non-working, and poor psychological health (ORga between 1.36 and 2.47). Overweight measured with BMIm was also related to insufficient income, poor physical health, and poor living environment (ORga between 1.38 and 1.54) unlike overweight measured with BMIsr which was also related to male gender, low school performance, tobacco and cannabis use, and being a victim of sexual abuse (ORga between 1.27 and 1.85). Obesity measured with BMIm and that measured with BMIrs were similarly associated with male gender, living with a single-parent, low school performance, having sustained violence, involvement in violence, poor physical health, poor psychological health, poor social relationships, and poor living environment (ORga between 1.55 and 2.96). Obesity measured with BMIm was also associated with being a victim of sexual abuse (ORga 2.22) unlike obesity measured with BMIsr which was also associated with having a father being a craftsman, tradesman, or firm head. Obesity measured with BMIm was also associated with having a father being a manual-worker or non-working (ORga 2.67 and 2.89, respectively) but clearly less strongly than obesity measured with BMIsr (ORga 3.67 and 5.06, respectively).

**Table 6:** Relationships of underweight, overweight, and obesity (vs. normal weight) assessed using self-reported and measured body mass index (BMIsr, BMIm) with various factors: odds ratio and 95% confidence interval

	**Overweight**				**Obese**			
	**BMIm**		**BMIsr**		**BMIm**		**BMIsr**	
	**ORga 95% CI**	**ORfm 95% CI**	**ORga 95% CI**	**ORfm 95% CI**	**ORga 95% CI**	**ORfm 95% CI**	**ORga 95% CI**	**ORfm 95% CI**
Boys	**1.17** 0.92-1.48	‒	1.27* 1.00-1.61	1.30* 1.03-1.65	1.59† 1.13-2.23	1.73† 1.21-2.47	1.73† 1.23-2.44	1.98† 1.37-2.86
Age (yr)	1.09 0.99-1.20	‒	1.09 0.99-1.20	‒	0.97 0.85-1.10	‒	0.92 0.80-1.05	‒
Family structure								
Intact	1.00	1.00	1.00	1.00	1.00	1.00	1.00	1.00
Parents divorced/separated and reconstructed family	1.24 0.94-1.64	‒	1.17 0.89-1.55	‒	1.03 0.69-1.53	‒	1.17 0.78-1.76	‒
Single parent and other situations	2.29‡ 1.57-3.37	1.89‡ 1.28-2.78	2.01‡ 1.40-2.89	1.70† 1.18-2.46	1.96† 1.17-3.31	‒	2.37‡ 1.46-3.86	1.85* 1.11-3.09
Father’s occupation								
Manager, professional, and intermediate professional	1.00	1.00	1.00	1.00	1.00	1.00	1.00	1.00
Craftsman, tradesman, and firm head	1.26 0.91-1.74	‒	1.23 0.89-1.70	‒	**0.80** 0.46-1.38	‒	1.72* 1.01-2.93	‒
Service worker and clerk	0.96 0.61-1.51	‒	0.93 0.59-1.45	‒	1.38 0.76-2.52	‒	1.65 0.84-3.21	‒
Manual worker and other occupations	1.50† 1.10-2.06	1.36* 1.02-1.81	1.36* 1.00-1.84	‒	*2.67*‡ 1.77-4.04	2.60‡ 1.79-3.77	*3.67*‡ 2.33-5.79	2.29‡ 1.56-3.38
Not working	2.47‡ 1.51-4.02	1.84* 1.13-3.00	2.13‡ 1.34-3.39	1.61* 1.02-2.55	*2.89*† 1.48-5.64	2.25* 1.18-4.33	*5.06*‡ 2.68-9.54	2.42† 1.31-4.50
Insufficient family income	1.38* 1.01-1.89	‒	**1.29** 0.95-1.75	‒	1.63* 1.07-2.47	‒	1.90† 1.27-2.85	‒
Low school performance (<10/20)	**1.49** 0.94-2.36	‒	**1.76**† 1.14-2.73	‒	2.51‡ 1.45-4.37	‒	2.96‡ 1.72-5.09	2.22† 1.24-3.97
Last-30 day substance use								
Tobacco	**1.46** 0.99-2.15	‒	**1.67**† 1.17-2.40	1.67† 1.16-2.38	1.49 0.87-2.55	‒	1.37 0.79-2.37	‒
Alcohol	1.03 0.80-1.34	‒	0.93 0.72-1.20	‒	0.88 0.60-1.27	‒	0.97 0.67-1.41	‒
Cannabis	**1.21** 0.72-2.04	‒	1.65* 1.03-2.64	‒	0.59 0.23-1.54	‒	0.37 0.11-1.22	0.23* 0.07-0.80
Hard drugs	1.58 0.77-3.22	‒	1.76 0.92-3.40	‒	1.10 0.37-3.32	‒	0.81 0.24-2.76	‒
Lack of regular physical/sports activity	0.99 0.67-1.45	‒	0.98 0.67-1.42	‒	0.94 0.54-1.62	‒	1.13 0.67-1.88	‒
Having sustained violence	1.10 0.87-1.40	‒	1.03 0.81-1.31	‒	1.76† 1.24-2.49	1.58† 1.11-2.27	1.68† 1.18-2.40	1.55* 1.08-2.24
Victim of sexual abuse	**1.58** 0.83-3.00	‒	1.85* 1.01-3.38	‒	2.22* 1.00-4.93	‒	**2.07** 0.91-4.70	‒
Involvement in violence	1.16 0.90-1.49	‒	1.18 0.92-1.53	‒	1.87‡ 1.28-2.73	‒	1.60* 1.10-2.33	‒
WHOQOL ≤25th percentile value								
Physical health	**1.54**† 1.16-2.05	1.45† 1.09-1.93	**1.25** 0.95-1.66	‒	1.85† 1.25-2.72	‒	1.76† 1.19-2.60	‒
Psychological health	1.52† 1.15-2.00	‒	1.37* 1.04-1.79	‒	2.51‡ 1.75-3.61	2.24‡ 1.55-3.26	2.24‡ 1.55-3.23	1.82† 1.24-2.69
Social relationships	1.28 0.98-1.68	‒	1.18 0.91-1.55	‒	1.73† 1.21-2.48	‒	1.55* 1.07-2.24	‒
Living environment	**1.50**† 1.14-1.98	‒	**1.18** 0.89-1.54	‒	2.15‡ 1.50-3.10	‒	1.85‡ 1.29-2.66	‒

*p < 0.05, †p < 0.01, ‡p < 0.001.

ORga: gender-age-adjusted odds ratio; ORfm: odds ratios adjusted for all factors (full model, retaining only significant factors (p < 0.05)).

Number of subjects : 1503 for BMIsr and 1436 for BMIm.

ORga for BMIm and BMIsr: in bold type values significant for one and non-significant for another; in italics both values were significant but they differed substantially.

Note: No significant factors were found for underweight defined by BMIsr and BMIm. They are not presented.

Table 6 shows that logistic regression models including all factors reveal that the main factors associated with overweight assessed with BMIsr were living with a single-parent, having a father being a manual worker or non-working, and poor physical health (odds ratios between 1.36 and 1.89) while those associated with overweight assessed with BMIm were male gender, living with a single-parent or a father being non-working, and tobacco use (between 1.30 and 1.70). Being obese assessed with BMIsr was associated with male gender, having a father who was a manual worker or non-working, having sustained violence, and poor physical health (between 1.58 and 2.60). Being obese assessed with BMIm was associated, in addition to these factors (between 1.55 and 2.42), with living with a single-parent (1.85), low school performance (2.22), and cannabis use (0.23).

## Discussion

This study among adolescents demonstrates that self-reported BMI was affected by under-reporting but also to a lesser degree by over-reporting, and that BMI measurement was more often refused than self-reported BMI. Our results also show that missingness of BMIsr and BMIm, as well as under and over-reporting, were not random, but were subject to error and artefact variously related to weight perception, and to socioeconomic, health-related, and behavioural factors. Our results confirm that, although self-reported weight and height overall exhibited small errors (small mean differences and intra-class correlation coefficients close to one) [7,18], they are unreliable in large population subgroups [17]. Our findings are original, as most studies have focused on gender, age, education, income, and ethnic group only [4,8,17]. They shed light on the considerable selection bias for studies on obesity and health outcomes in adolescent populations. Analysing BMI category rather than BMI as continuous variable appeared to be appropriate and to give results of interest.

In line with other studies [4,9], our results reveal that about 20% of BMIsr were affected by under-reporting, over-reporting (with changes in BMI category) or reporting refusal (11.8%, 6.0%, and 3.6%). We found that BMI measurement had the disadvantage as it was twice as often refused as self-reporting. We also noted that measurement refusal was related to more numerous covariates. First, both self-reporting and measurement refusals were related to living with a single-parent, low school performance, lack of physical/sports activity, poor psychological health, and feeling much too fat. In addition to these factors, measurement refusal was also related to older age, having divorced/separated parents or reconstructed families, having a father being a manual-worker or non-working, insufficient family income, tobacco/cannabis use, involvement in violence, poor physical health, and poor living environment. However, reporting refusal was also associated with having sustained violence, poor social relationships, and feeling too thin. These original results point to the strong biases resulting from a wide range of vulnerability factors related to weight, socioeconomic features, unhealthy behaviours, and health outcomes. It can be noted that logistic regression models including all factors (i.e. taking account of the interdependences of various factors) retained clearly different factors: having sustained violence, and feeling much too thin for self-reporting refusal; age, living with a single-parent, low school performance, lack of physical/sports activity, and poor psychological health. This suggests that self-reporting and measurement refusals reflect different individual features that could inform investigators and carers using self-reported or measured BMIs.

Some studies concluded to an under-reporting of BMIsr compared to BMIm when both were considered as continuous variables (disregarding refusals) [4,40]. If our analysis focused on BMIsr and BMIm as continuous variables we could conclude to BMIsr under-reporting, as the mean value of BMIm-BMIsr was positive although under-reporting was twice as common as over-reporting. In a study among adults, Brestoff et al. defined accurately reported, under-reported, over-reported weight (height) according to whether or not the difference with measured values exceeded 2.0 kg (2.0 cm) [40]. These threshold values seem rather arbitrary when we consider the large inter-individual variations of weight/height in various populations, and the gender difference for example. The choice is problematic for adolescents in a rapid growth period where threshold values used in the literature for underweight, overweight and obesity vary with gender and age [1,37]. Therefore we used recommended cut-offs for French adolescents [37] and defined under or over-reporting when using BMIm and BMIsr resulted in different BMI categories. Our choice was however also arbitrary, but our results suggest that analysing BMIm and BMIsr as continuous variables may not be relevant because the difference between them was rather small for most adolescents and the main problem concerned 16.6% of subjects (232 among 1401 subjects, Table 2) classified differently as underweight, normal, overweight, or obese with BMIm and BMIsr. This discrepancy results in misclassification for many subjects when using BMIsr. This was attested by the low kappa coefficients (about 70%).

An important finding is that a number of potential socioeconomic, health-related, and behavioural factors were substantially and differently associated with overweight and obesity assessed using BMIsr and BMIm. Interestingly, social disparities in obesity were much stronger with BMIsr than with BMIm, but this difference was not observed for overweight. The covariates investigated were generally much more strongly related to obesity than to overweight whether assessed with BMIsr or BMIm. Furthermore overweight assessed with BMIsr and BMIm yielded more discrepancies than obesity for associations with covariates. This finding could suggest that overweight, covering a wide range of intermediate BMI values, was more difficult to be perceived than obesity by some adolescents, especially by boys and those with low school performance, tobacco or cannabis use, or having been a victim of sexual abuse. But, BMIm appeared to be more relevant than BMIsr to evaluate the associations of overweight with insufficient family income, poor physical health, and poor living environment. Caution needs thus to accompany the conclusions that can be drawn. Himes recommends that self-reported height and weight should only be used with caution and cognizance of limitations, biases, and uncertainties [15]. In the Brener et al. adolescent study [11] the sensitivity and specificity of BMIsr for identifying overweight subjects were 60.5% and 98.0%, and for identifying obese subjects they were 54.9% and 99.2%, respectively. Thus as few as 55% (positive predictive value) of those who are truly overweight would be correctly identified as such using BMIsr. Results from other studies are not more encouraging [7].

We found that (after adjustment for gender and age) underweight (assessed with BMIm) was associated with a 5-fold greater likelihood of BMI over-reporting, and that overweight/obesity was linked to an 11-fold likelihood of BMI under-reporting, in line with the literature [7,9-16]. Furthermore these risks changed little with further adjustment for socioeconomic, health-related, and behavioural factors. These tendencies observed beyond individual and socioeconomic features suggest a common problem among adolescents in a rapid growth period. This invites parents, physicians, and schools to allow to students regular BMI assessment.

Interestingly both under and over-reporting were found to be more common among boys, subjects with poor psychological health, or feeling too fat. These results suggest that these factors were associated with a lack of accuracy (in both directions) for self-reported values. Under-reporting was found to be related to involvement in violence and over-reporting to younger age and alcohol consumption. The role of covariates is not well documented in the literature. A study among Australian adults found that major determinants of reporting error were age, gender, measured BMI, and education [4]. Shiely et al. stated that using BMIsr leads to underestimation of obesity prevalence in the population and this error increased with time, possibly because of BMI variations across time [41]. We think that temporal variations in socioeconomic, health-related, and behavioural factors in our society may play a prominent role in weight perception, weight-related issues, and the desire and ability to monitor body weight.

In line with the literature [10,11,16] the influences of weight perception on self-reported data were confirmed. For better understanding let us here examine the relationships observed with the study covariates. We found that feeling too fat or too thin correlated with poor psychological health and suicidal ideation (result not shown). This confirms the results in other populations [28-30]. Importantly our study reveals new findings that feeling too fat and feeling too thin were linked to a wide range of socioeconomic and individual factors. First, as reported by other studies [7,31] feeling too fat affected girls, overweight and obese subjects (assessed with BMIm) more markedly, while feeling too thin affected boys and underweight subjects (assessed with BMIm) more. Second, both feeling too fat and feeling too thin were linked to similar problems: low school performance, poor physical health, poor psychological health, and poor living environment, and being victim of sexual abuse. The relationship between feeling too fat and poor quality of life is known [31]. Exposure to these problems may result in greater stress which is associated with a greater drive to eat, including feelings of disinhibited eating, binge eating, hunger, and more ineffective attempts to control eating [42] leading to dissatisfaction or inaccurate weight perception (in the two directions, too fat or too thin). Our study shows that, unlike feeling too thin, feeling too fat also had a high socioeconomic component in its strong associations with living with a single parent, father’s occupation, and insufficient family income [31]. Interestingly, feeling too fat was also related to tobacco and hard drug use, having sustained violence, involvement in violence, and poor social relationships. These findings were expected because these social/material deprivations are linked to unhealthy diet, poor physical activity, poor physical/mental health, and poor living conditions [23,35]. We did not find a link between lack of regular physical/sports activity and feeling too fat or feeling too thin. This could be explained by the compulsory activities at school. So feeling too fat and feeling too thin can result in a number of problems that severely affect adolescent health and school achievement. These findings call for adolescent-centred prevention involving the adolescents themselves, their families, physicians, and schools.

Another important finding is that feeling too fat was associated with a greater likelihood of reporting refusal than of measurement refusal, and of over-reporting than of under-reporting. However, the likelihood was highly exacerbated for over-reporting but became non-significant after controlling for all socioeconomic, health-related, and behavioural covariates (and BMIm, except for measurement refusal). Regarding feeling too thin, after controlling for BMIm, it was associated with a higher likelihood of reporting refusal only and this was less marked after controlling for all socioeconomic, health-related, and behavioural covariates; feeling too thin was not associated with under-reporting but the association became highly significant after controlling for all covariates. Thus BMIsr tended to be overestimated by the subjects feeling too fat and underestimated by those feeling too thin. The feeling-too-fat – reporting refusal association became non-significant after controlling for socioeconomic, health-related, and behavioural covariates (contribution 44%). The feeling-too-fat – under-reporting association was the reverse (adjusted OR < 1). Finally the associations between feeling too fat and over-reporting and between feeling too thin and under-reporting were reinforced by covariates (negative contributions −82% and −17%, respectively). These risk patterns point to the large and very different roles of socioeconomic, health-related and behavioural covariates in weight perception, denial of underweight, denial of overweight/obesity, and in self-reported data. This issue did not affect measurement refusal; BMIm was thus little influenced by weight perceptions.

Finally, should studies only use measurement, or also use BMIsr to complete missing BMIm? Further analysis shows that among the 123 subjects with missing BMIm, BMIsr were available for 102 subjects, leaving only 21 subjects (1.3% of the total sample) with missing values. We found that the 102 subjects fell into similar BMIsr categories to the subjects with available BMIm (p = 0.21 with inclusion of missing BMIsr category, and p = 0.35 with its exclusion). Belonging to this group was significantly associated (p < 0.05) with older age (ORga 1.33), living with a single-parent (3.08), being the child of a manual-worker (1.76), having a father being non-working (3.22), insufficient family income (1.67), low school performance (2.72), tobacco use (2.02), hard drugs use (2.49), lack of physical/sports activity (2.34), involvement in violence (1.68), poor physical health (1.77), poor psychological health (2.00), and poor living environment (2.21). It was not surprising that the risk factor were fairly similar to those for measurement refusal. We may thus suggest collecting and using self-reported values to replace missed measured values.

### Limitations and strengths

Some methodological aspects warrant comments. First, the study was based on self-reported data, but self-administered anonymous questionnaires are widely used and arguably good tools to study adolescent living conditions, mental health, and unhealthy behaviours [23,25,26]. Second, the adolescents were aware that there would be measurements after self-reporting. Some studies introduced a time lapse (up to several weeks) between self-reporting and measurement [17] making the data subject to time variations. We preferred to perform them at the time of the survey. In some studies participants were also aware that they were to undergo measurements after questionnaire completion [9,41]. Although knowledge of impending measurement could lead to more accuracy in self-report, it is believed to play a small role [41]. It can be noted that the intra-class correlation coefficients found between reported and measured height, weight, and BMI were close to those in other adolescent studies [15,18]. Third, our results should be interpreted with prudence because of the small numbers of subjects, especially for reporting refusal. Fourth, given the large number of statistical tests performed, type I error may be a concern, but most tests were significant at the 0.001 level, with very high odds ratio estimates.

Strengths of the study also deserve to be mentioned. The participation rate was high (94%). The data collection and weight and height measurements were undertaken by the same trained physician over a short period (May-June 2010) to avoid inter-observer and seasonal variations. The prevalences of a wide range of health/behaviour outcomes assessed using the same measures were similar to those of a representative sample of adolescents in France [25].

## Conclusion

Our study demonstrates that BMI self-reporting meets with refusals linked to a number of factors: living with a single-parent, low school performance, lack of physical/sports activity, having sustained violence, poor psychological health, poor social relationships, and feeling too thin or too fat. Self-reported BMIs should be used cautiously because they were strongly affected by under-reporting, which was related to numerous factors: male gender, involvement in violence, poor psychological health, overweight/obesity (assessed by BMI measurement), and feeling too fat. Self-reporting was also strongly affected by over-reporting which was related to male gender, age, alcohol use, underweight (assessed by BMI measurement), and feeling too fat. Our work also recommends prudence when using measured BMIs, as measurement was more often refused than self-report of BMIs. In addition to risk factors for self-reporting (except having sustained violence and poor social relationships), measurement refusal was linked to several other covariates: older age, living with divorced/separated parents, having a father being a manual-worker or non-working, insufficient family income, tobacco/cannabis use, involvement in violence, poor physical health, and poor living environment. The contributions of socioeconomic, health-related, and behavioural factors to the associations of feeling too fat or feeling too thin with reporting refusal, under-reporting and over-reporting, ranged from −82% to 44%. Identifying risk factors for overweight and obesity assessed with self-reported or measured BMIs resulted in substantial discrepancies, and this calls for caution in matters of prevention and care. Self-reporting and measurement are thus affected by numerous biases, mostly related to vulnerabilities, which are well known as potential risk factors for obesity. Finally, preference should be given to BMI measurement, and our findings suggest that everything should be done to reduce measurement refusal among vulnerable subjects. When BMI measurement cannot be performed, refusals also need to be reduced in self-reporting, as does under and over-reporting among vulnerable adolescents. Our results may also suggest that socioeconomic, health-related, and behavioural factors could be taken into account to estimate true value of BMI from self-reported BMI. Further research in different populations is needed.

## Competing interest

The authors declare that they have no competing interest.

## Authors’ contributions

NC designed the survey, carried out the study and had the main responsibility for writing the manuscript. KC designed the survey, carried out the study and participated in writing the manuscript. AM, MB, SL and BF participated in designing the study and writing the manuscript. All authors read and approved the final manuscript.

## Pre-publication history

The pre-publication history for this paper can be accessed here:

http://www.biomedcentral.com/1471-2458/13/815/prepub

## Supplementary Material

Additional file 1 **Comparison between the study population and France (ESPAD survey **[25,26]**) (%).** Click here for file

Additional file 2 Relationships of body image perception with self-reported or measured BMIs:%. Click here for file

Additional file 3 Relationships between feeling too fat or too thin (vs. right weight) and various factors: gender-age-adjusted odds ratio and 95% confidence interval. Click here for file
