# Supplementary material for: Self-reporting and measurement of body mass index in adolescents: refusals and validity, and the possible role of socioeconomic and health-related factors
Source: BMC Public Health. 2013 Sep 8;13:815. doi: 10.1186/1471-2458-13-815 (PMC3846114; doi:10.1186/1471-2458-13-815)
Supplement: Additional file 1 — Comparison between the study population and France (ESPAD survey [25,26]) (%). [file 1471-2458-13-815-S1.doc]

**Additional file 1. Comparison between the study population and France (ESPAD survey [25, 26**]) (%)

|  | Study population  (limited to <16 years ^a^)  (n=1,524) | France  (ESPAD survey)  (<16 years) |
| --- | --- | --- |
| *Number of subjects* | *1,524* | *8,367* |
| Boys | 49.9 | 48.9 |
| Family structure |  |  |
| Intact | 63.2 | 74.7 |
| Reconstructed | 15.0 | 11.3 |
| Single parent | 16.4 | 11.7 |
| Others | 5.4 | 2.3 |
| Obese (with self-reported data) | 10.6 | 6.9 |
| Last-30-day substance use |  |  |
| Tobacco | 10.7 | 13.6 |
| Alcohol | 34.7 | 34.6 |
| Cannabis | 5.1 | 5.5 |
| Sleep disorders | 32.6 | 29.0 |
| Asthma | 17.2 | 16.3 |
| Depressive symptoms | 13.1 | 9.8 |
| Last-12-month suicide ideation | 11.6 | 9.1 |
| Lifetime suicide attempt | 9.6 | 7.2 |
| Sexual abuse | 3.4 | 1.9 |
| Having sustained violence | 53.3 | 51.5 |
| Involvement in violence | 59.1 | 64.7 |

^a^ were excluded 35 subjects aged 16 years or over.
