# Supplementary material for: Self-reporting and measurement of body mass index in adolescents: refusals and validity, and the possible role of socioeconomic and health-related factors
Source: BMC Public Health. 2013 Sep 8;13:815. doi: 10.1186/1471-2458-13-815 (PMC3846114; doi:10.1186/1471-2458-13-815)
Supplement: Additional file 2 — Relationships of body image perception with self-reported or measured BMIs:%. [file 1471-2458-13-815-S2.doc]

Additional file 2. Relationships of body image perception with self-reported or measured BMIs: %

|  |  | Body image perception | | | | |
| --- | --- | --- | --- | --- | --- | --- |
|  | Number of subjects | Right weight | Much too thin | A bit too thin | A bit too fat | Much too fat |
| Self-reported BMI |  |  |  |  |  |  |
| Underweight | 39 | 38.5 | 7.7 | 53.8 | 0 | 0 |
| Normal | 893 | 67.0 | 1.5 | 14.8 | 15.1 | 1.7 |
| Overweight | 392 | 44.6 | 0.8 | 1.0 | 49.0 | 4.6 |
| Obese | 156 | 16.0 | 0 | 1.3 | 60.3 | 22.4 |
| Measured BMI |  |  |  |  |  |  |
| Underweight | 19 | 36.8 | 5.3 | 57.9 | 0 | 0 |
| Normal | 840 | 66.0 | 1.9 | 16.7 | 14.2 | 1.3 |
| Overweight | 390 | 48.2 | 0.5 | 1.3 | 45.1 | 4.9 |
| Obese | 165 | 15.2 | 0.6 | 0 | 64.2 | 20.0 |

BMI: Body mass index.
