# Supplementary material for: Self-reporting and measurement of body mass index in adolescents: refusals and validity, and the possible role of socioeconomic and health-related factors
Source: BMC Public Health. 2013 Sep 8;13:815. doi: 10.1186/1471-2458-13-815 (PMC3846114; doi:10.1186/1471-2458-13-815)
Supplement: Additional file 3 — Relationships between feeling too fat or too thin (vs. right weight) and various factors: gender-age-adjusted odds ratio and 95% confidence interval. [file 1471-2458-13-815-S3.doc]

Additional file 3. Relationships between feeling too fat or too thin (vs. right weight) and various factors: gender-age-adjusted odds ratio and 95% confidence interval

|  | Too fat (vs. feeling the right weight) | |  | Too thin (vs. feeling the right weight) | |
| --- | --- | --- | --- | --- | --- |
|  | ORga 95% CI | ORfm 95% CI |  | ORga 95% CI | ORfm 95% CI |
| *Number of subjects* | *1,344* |  |  | *1,021* |  |
| Boys | 0.49‡ 0.39-0.62 | 0.33‡ 0.25-0.44 |  | 1.40* 1.01-1.93 | 1.85‡ 1.31-2.60 |
| Age (yr) | 1.08 0.98-1.18 | ‒ |  | 0.98 0.87-1.11 | ‒ |
| Family structure |  |  |  |  |  |
| Intact | 1.00 | 1.00 |  | 1.00 | 1.00 |
| Parents divorced/separated and reconstructed family | 1.31* 1.01-1.70 | ‒ |  | 1.06 0.73-1.54 | ‒ |
| Single parent and other situations | 1.47* 1.04-2.08 | ‒ |  | 0.86 0.50-1.48 | ‒ |
| Father’s occupation |  |  |  |  |  |
| Manager, professional, and intermediate professional | 1.00 | 1.00 |  | 1.00 | 1.00 |
| Craftsman, tradesman, and firm head | 1.32 0.96-1.80 | ‒ |  | 1.10 0.71-1.69 | ‒ |
| Service worker and clerk | 1.50 0.99-2.27 | ‒ |  | 1.35 0.78-2.34 | ‒ |
| Manual worker and other occupations | 1.81‡ 1.36-2.42 | ‒ |  | 1.30 0.86-1.96 | ‒ |
| Not working | 1.76† 1.13-2.74 | ‒ |  | 1.08 0.55-2.14 | ‒ |
| Insufficient income | 1.43* 1.07-1.92 | ‒ |  | 1.46 0.97-2.19 | ‒ |
| Low school performance (<10/20) | 2.73‡ 1.80-4.16 | 1.81* 1.11-2.94 |  | 2.20† 1.23-3.93 | 2.21† 1.19-4.08 |
| Last-30 day substance use |  |  |  |  |  |
| Tobacco | 2.07‡ 1.45-2.95 | ‒ |  | 1.50 0.90-2.52 | ‒ |
| Alcohol | 1.15 0.91-1.47 | ‒ |  | 0.87 0.61-1.23 | ‒ |
| Cannabis | 1.46 0.90-2.38 | ‒ |  | 1.19 0.59-2.41 | ‒ |
| Hard drugs | 2.14* 1.09-4.23 | ‒ |  | 1.93 0.77-4.82 | ‒ |
| Lack of regular physical/sports activity | 1.27 0.90-1.79 | ‒ |  | 1.02 0.61-1.69 | ‒ |
| Having sustained violence | 1.37† 1.09-1.72 | ‒ |  | 1.01 0.74-1.40 | ‒ |
| Victim of sexual aggression | 3.31‡ 1.76-6.25 | ‒ |  | 2.74* 1.17-6.40 | ‒ |
| Involvement in violence | 1.35* 1.06-1.72 | ‒ |  | 0.76 0.55-1.07 | ‒ |
| WHOQOL <25^th^ percentile value |  |  |  |  |  |
| Physical health | 2.25‡ 1.72-2.93 | ‒ |  | 1.68† 1.14-2.46 | ‒ |
| Psychological health | 3.61‡ 2.79-4.67 | 3.17‡ 2.36-4.26 |  | 2.00‡ 1.38-2.90 | 1.84† 1.24-2.73 |
| Social relationships | 1.61‡ 1.25-2.06 | ‒ |  | 1.03 0.71-1.50 | ‒ |
| Living environment | 2.24‡ 1.74-2.90 | ‒ |  | 1.56* 1.08-2.25 | ‒ |
| Measured body mass index (BMIm) |  |  |  |  |  |
| Underweight | 0 | ‒ |  | 6.66‡ 2.55-17.4 | 7.13‡ 2.69-18.9 |
| Normal weight (reference) | 1.00 | 1.00 |  | 1.00 | 1.00 |
| Overweight | 5.39‡ 4.01-7.25 | 5.18‡ 3.82-7.03 |  | 0.12‡ 0.05-0.25 | 0.12‡ 0.05-0.26 |
| Obese | 36.9‡ 22.4-60.7 | 33.2‡ 20.0-55.1 |  | 0.13* 0.02-0.97 | 0.11* 0.01-0.84 |
| Refusal | 3.74‡ 2.37-5.89 | 2.94‡ 1.83-4.74 |  | 0.88 0.48-1.64 | ‒ |

*p<0.05, †p<0.01, ‡p<0.001.

ORga: gender-age-adjusted odds ratio.

ORfm: odds ratios adjusted for all factors (full model, retaining only significant factors (p<0.05)).
